# 

**Published:** 1889-11

**Authors:** 


Now is the time to renew your Subscription.

VOL. X. NOVEMBER, 1889. No. 11.

LIBRARY
Indexed
S. G. O.

### THE International Dental Journal

A MONTHLY PERIODICAL

DEVOTED TO

### DENTAL AND ORAL SCIENCE.

W. XAVIER SUDDUTH, M.D., D.D.S.,
EDITOR.
Office, 716 Filbert Street, Philadelphia.

PUBLISHED BY THE

INTERNATIONAL DENTAL PUBLICATION COMPANY,

51 WEST THIRTY-SEVENTH STREET, NEW YORK CITY,
—AND—
716 FILBERT STREET, PHILADELPHIA.

ENTERED AT PHILADELPHIA POST-OFFICE AS SECOND-CLASS READING MATTER.

$2.50 per Annum, in Advance. Single Copies, 25 Cents.

PRINTED BY J. B. LIPPINCOTT COMPANY, PHILADELPHIA.

